# Chromokinesin KIF4A teams up with stathmin 1 to regulate abscission in a SUMO-dependent manner

**DOI:** 10.1242/jcs.248591

**Published:** 2020-07-24

**Authors:** Sabine A. G. Cuijpers, Edwin Willemstein, Jan G. Ruppert, Daphne M. van Elsland, William C. Earnshaw, Alfred C. O. Vertegaal

**Affiliations:** 1 Cell and Chemical Biology, Leiden University Medical Center, 2333 ZA Leiden, The Netherlands; 2 Wellcome Centre for Cell Biology, University of Edinburgh, EH9 3JR Edinburgh, Scotland, UK

**Keywords:** Mitosis, Abscission, Cytokinesis, KIF4A, Post-translational modification, SUMO, Stathmin 1

## Abstract

Cell division ends when two daughter cells physically separate via abscission, the cleavage of the intercellular bridge. It is not clear how the anti-parallel microtubule bundles bridging daughter cells are severed. Here, we present a novel abscission mechanism. We identified chromokinesin KIF4A, which is adjacent to the midbody during cytokinesis, as being required for efficient abscission. KIF4A is regulated by post-translational modifications. We evaluated modification of KIF4A by the ubiquitin-like protein SUMO. We mapped lysine 460 in KIF4A as the SUMO acceptor site and employed CRISPR-Cas9-mediated genome editing to block SUMO conjugation of endogenous KIF4A. Failure to SUMOylate this site in KIF4A delayed cytokinesis. SUMOylation of KIF4A enhanced the affinity for the microtubule destabilizer stathmin 1 (STMN1). We here present a new level of abscission regulation through the dynamic interactions between KIF4A and STMN1 as controlled by SUMO modification of KIF4A.

## Abstract (precis)

**Summary:** A new mechanism of abscission regulation is identified through the dynamic interactions between KIF4A and stathmin 1 as controlled by SUMO modification of KIF4A.

## INTRODUCTION

Abscission is the final step of cell division in which the two daughter cells are separated to start their own independent life. Our present understanding of abscission is limited (Mierzwa and Gerlich, 2014). Prior to abscission, the cleavage furrow is formed involving key players, like actin, myosin, septin and anillin, culminating in the formation of the constricted intercellular bridge (Field and Alberts, 1995; Joo et al., 2007; Lewellyn et al., 2011; Mabuchi, 1994; Mavrakis et al., 2014; Mullins and Biesele, 1977; Nunnally et al., 1980; Reichl et al., 2008; Sanger et al., 1994; Schroeder, 1968; Uehara et al., 2010; Zhou and Wang, 2008). Subsequently, the cortex of the intercellular bridge is narrowed to a single stalk of 17-nm-diameter filaments (Guizetti et al., 2011). This process involves the ESCRT-III complex and spastin-mediated microtubule (MT) disassembly (Connell et al., 2009; Yang et al., 2008). Although spastin knockdown results in a delay in MT disassembly, the process is not abolished, indicating the existence of additional abscission pathway components.

Post-translational modifications play a major role in mitotic regulation. Important modifications include phosphorylation and modification by small proteins including ubiquitin and small ubiquitin-like modifier (SUMO) (Barr et al., 2004; Cubeñas-Potts et al., 2015; Dasso, 2008; Deribe et al., 2010; Eifler and Vertegaal, 2015; Flotho and Melchior, 2013; Gareau and Lima, 2010; Glotzer et al., 1991; Gurley et al., 1973; King et al., 1994, 1996; Li and Hochstrasser, 1999; Peters, 2002; Ribet et al., 2017; Pohl and Jentsch, 2008; Seeler and Dejean, 2017; Seufert et al., 1995; Teixeira and Reed, 2013; Zhang et al., 2008). Inactivating SUMO conjugation machinery components indeed delays cell cycle progression and results in chromosomal aberrations (Hayashi et al., 2002; He et al., 2015; Nacerddine et al., 2005; Schimmel et al., 2014), but it is unclear how. SUMO (herein referring to mammalian SUMO1-SUMO3) is conjugated to hundreds of substrates (Hendriks et al., 2014; Hendriks and Vertegaal, 2016a; Schimmel et al., 2014), but little is known about the impact of SUMO on key substrates in mitosis. What is observed is that SUMO accumulates at the midbody during cytokinesis, but – again – its functional relevance nor the relevant substrates for cytokinesis are unclear (Fu et al., 2005; Nie and Boddy, 2016; Pelisch et al., 2017). One protein critical for cytokinesis is the chromokinesin KIF4A, which is preferentially localized at the midbody, where it is important for PRC1 recruitment and for MT organization at the midbody (Hu et al., 2011; Kurasawa et al., 2004; Lee and Kim, 2004; Zhu and Jiang, 2005; Zhu et al., 2006). Here, we identified KIF4A as a SUMOylated protein and show that this modification is important for dynamic interaction with the MT destabilizer stathmin 1 (STMN1). SUMOylation thus has a role in cytokinesis.

## RESULTS

### The human chromokinesin KIF4A is modified by one SUMO moiety

**Fig. 1.**
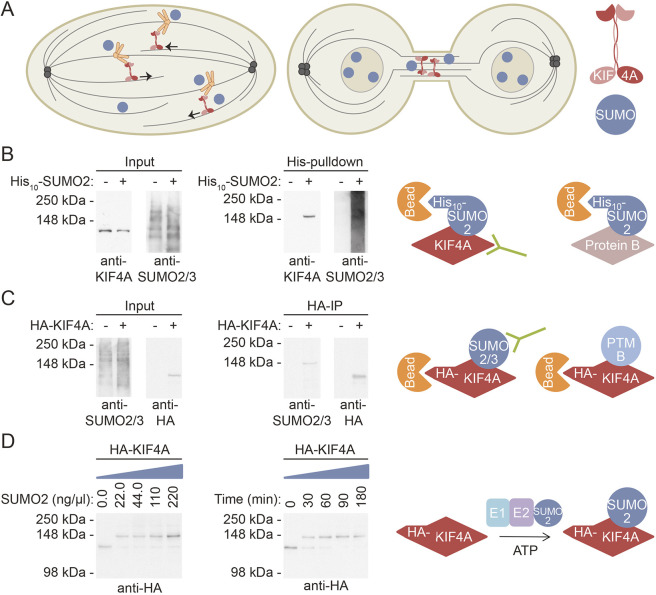
**The human chromokinesin KIF4A is modified by a single SUMO moiety.** (A) Chromokinesin KIF4A is required for the positioning of mitotic chromosomes and stabilization of the bipolar spindle. In cytokinesis, KIF4A localizes at the intercellular bridge. The post-translational modifier SUMO is required for mitosis, and is conjugated to KIF4A. In this project, we investigate the functional role of KIF4A SUMOylation. (B) U2OS cells without or with stable expression of His_10_–SUMO2 were lysed. A His_10_ pulldown was performed to enrich for SUMOylated proteins. Input and pulldown samples were analyzed by immunoblotting using antibodies against KIF4A and SUMO2/3. (C) U2OS cells were transfected with a control or HA–KIF4A WT construct and lysed after 3 days. An HA IP was performed to enrich HA-KIF4A WT. Input and IP samples were analyzed by immunoblotting using antibodies against SUMO2/3 or the HA tag. (D) U2OS cells were transfected with a construct encoding HA–KIF4A WT, lysed after 3 days and an HA IP was performed. The purified HA-KIF4A WT was *in vitro* SUMOylated by the addition of SUMO E1 and SUMO E2, and either incubated at 4°C for 3 h with the indicated concentrations of SUMO2 (left) or for the indicated time with 220 ng/µl SUMO2 (right). Samples were analyzed by immunoblotting using an antibody against the HA-tag. The experimental procedures for B–D are summarized in the cartoons on the right. Each experiment was performed at least three times.

We aimed to understand the mechanisms and effects of SUMOylation of the chromokinesin KIF4A (Cubeñas-Potts et al., 2015; Kurasawa et al., 2004) to obtain more detailed understanding of how SUMO regulates this important process of mitosis (Fig. 1A). KIF4A has been identified as SUMO target protein in at least ten individual SUMOylation proteomics studies, indicating that this protein is one of the top targets for SUMO (Hendriks et al., 2014; Hendriks and Vertegaal, 2016a; Schimmel et al., 2014). Immunoblot analysis confirmed KIF4A as a SUMO target. To enrich for SUMOylated proteins, U2OS cell lines either stably expressing His_10_–SUMO2 or not at close to endogenous levels were lysed and a His_10_ pulldown was performed. Immunoblot analysis revealed modification of endogenous KIF4A by one single SUMO moiety (Fig. 1B). This was confirmed by transfecting U2OS cells with either control or HA–KIF4A constructs. After enrichment for KIF4A by HA immunoprecipitation (IP), under conditions where SUMOylation is preserved, immunoblotting against SUMO2/3 confirmed modification of HA–KIF4A by endogenous SUMO2/3 (Fig. 1C). Additionally, HA–KIF4A enriched in a non-denaturing manner was able to be SUMOylated *in vitro* upon addition of SUMO E1 (SAE1/2), SUMO E2 (UBC9) and SUMO2 (Fig. 1D).

While KIF4A was efficiently modified by SUMO under *in vitro* conditions, the level of SUMOylation was considerably lower in tissue culture cells as the SUMOylated fraction of KIF4A could not be observed by staining for bulk KIF4A in input samples. The modification might be specific for a certain cell cycle phase or for a functionally distinct fraction of KIF4A. However, no clear dynamic SUMOylation levels for KIF4A were observed throughout the cell cycle (Fig. S1) or during mitosis (Fig. S2). This suggests that either the time window during which the fraction of SUMOylated KIF4A shows dynamics is too small to resolve using this experimental set-up, or that a specific fraction of SUMOylated KIF4A is continuously present.

### KIF4A is SUMOylated on lysine 460 in a SIM-dependent manner

**Fig. 2.**
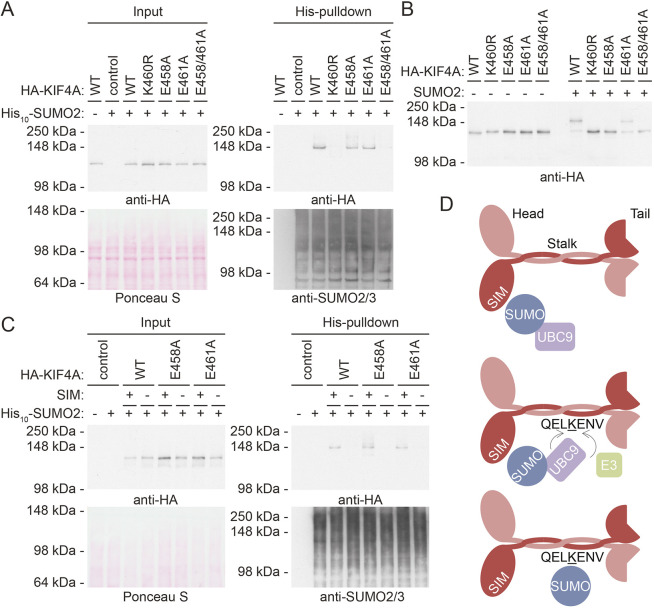
**KIF4A is SUMOylated on lysine 460 in a SIM-dependent manner.** (A) U2OS cells without or with stable expression of His_10_–SUMO2 were transfected with a control, HA–KIF4A WT or indicated mutant construct and lysed after 3 days. A His_10_ pulldown was performed to enrich for SUMOylated proteins. The samples were analyzed by immunoblotting using antibodies against the HA tag and SUMO2/3, while equal loading was confirmed by Ponceau S staining. (B) U2OS cells were transfected with plasmids encoding HA–KIF4A WT or the indicated mutants, lysed after 3 days, and the HA-tagged proteins were enriched by IP. An *in vitro* SUMOylation assay was performed by the addition of SUMO E1 and SUMO E2, followed by incubation for 3 h at 4°C in the presence of 220 ng/µl SUMO2. Samples were analyzed by immunoblotting using an antibody against the HA tag. (C) U2OS cells without or with stable expression of His_10_–SUMO2 were transfected with control plasmid, or plasmids encoding HA–KIF4A WT, HA–KIF4A E458A or HA–KIF4A E461A that either did not or did contain additional mutations to abolish the SUMO interaction motif (SIM ILDLL mutated to AADAA). After 3 days, cells were lysed. Upon enrichment for SUMOylated proteins by His_10_ pulldown, samples were analyzed by immunoblotting with antibodies against the HA tag or SUMO2/3, and by Ponceau S staining to confirm equal loading. Each experiment was performed at least three times. (D) A cartoon depicting the proposed mechanism of KIF4A SUMOylation in human cells. The SUMO E2 (UBC9)–SUMO complex interacts with the KIF4A dimer via the SIM in its head domain. Subsequently, UBC9 is able to covalently attach SUMO to lysine 460 in the stalk domain, either directly through the inverted consensus motif (ExK) or with the help of a SUMO E3 ligase through the second motif (KE).

Modification by one single SUMO2 moiety indicates targeting of a particular lysine residue in KIF4A. Various lysine-to-arginine KIF4A mutants were made to localize the SUMO acceptor lysine in KIF4A and transfected into U2OS cell lines without or with stable expression of His_10_–SUMO2. Mutating lysine 460 to arginine (K460R) abolished HA–KIF4A SUMOylation (Fig. 2A). The SUMO E2 UBC9 reportedly recognizes the consensus SUMOylation motif KxE in target proteins (Bernier-Villamor et al., 2002). Although KIF4A lysine 460 is not located in this specific motif, two adjacent glutamic acid residues could be part of either an inverted consensus motif ExK (Matic et al., 2010) or a less common KE motif (Pichler et al., 2005). While the single motif mutations did not abolish HA–KIF4A SUMOylation, replacing both glutamic acid residues simultaneously did, suggesting that both motifs can be utilized by the SUMO conjugation machinery in cells in a redundant manner.

To verify these results, an *in vitro* SUMOylation assay was performed on purified wild-type (WT) HA–KIF4A and mutant HA–KIF4A proteins. While WT HA-KIF4A was SUMOylated upon addition of SUMO2, both the K460R and E458A/E461A mutants were not, confirming the results obtained from cells (Fig. 2B). However, unlike our observation in cells, mutating the inverted consensus motif was sufficient to abolish KIF4A SUMOylation *in vitro*. The *in vitro* assay only contained SUMO E1 and SUMO E2 enzymes. However, cells express various SUMO E3 proteins that can contribute to the specific modification of KIF4A. Therefore, our experiments suggest that KIF4A SUMOylation occurs via two complementary mechanisms either through direct recognition of the inverted consensus motif by UBC9 or supported by a SUMO E3 to recognize the KE motif.

Interestingly, in-depth analysis of the sequence of KIF4A also revealed a potential SUMO interaction motif (SIM) at amino acids (aa) 148–152, located within the motor domain of this kinesin family member. To determine whether the SIM influences SUMOylation of KIF4A, this motif was separately mutated from ILDLL to AADAA to keep the hydrophobic nature of the amino acids, but to prevent SUMO interaction according to common practice in the SUMO field (Song et al., 2004). U2OS cells either stably expressing His_10_–SUMO2 or not were transfected with WT or mutant HA–KIF4A plasmids. Upon His_10_ pulldown and immunoblot analysis, the SIM mutation was observed to abolish SUMOylation of each of these three HA–KIF4A constructs (Fig. 2C). Thus, KIF4A SUMOylation requires this SIM. Therefore, we propose that a SUMO–UBC9 complex is able to bind the SIM in KIF4A, which brings the SUMO E2 in close proximity to lysine 460 to either directly recognize the inverted consensus motif or SUMOylate KIF4A with the help of a SUMO E3 (Fig. 2D).

**Fig. 3.**
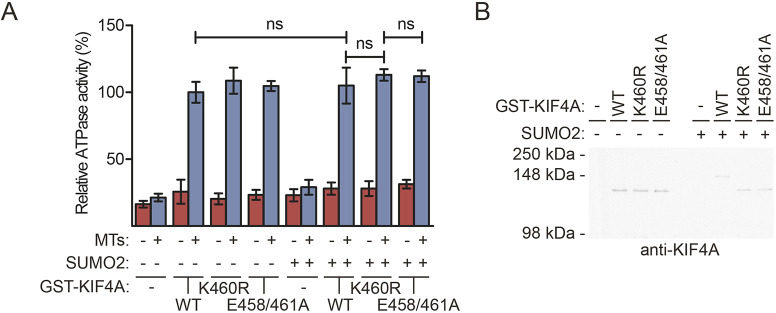
**SUMOylation does not affect ATPase activity of KIF4A.** (A) *In vitro* SUMOylation reactions were performed on negative control samples and samples containing wild-type (WT) or SUMOylation-deficient (K460R and E458A/E461A) recombinant GST–KIF4A. Additional negative control assays were carried out in the absence of SUMO2 as indicated. Subsequently, an ATPase activity assay was performed in the absence or presence of MTs and absorbance was measured. The mean±s.d. percentage of relative ATPase activity for three independent experiments is shown. A two-sided Student's *t*-test was used to calculate *P*-values and the difference was considered not significant (ns) when *P*>0.05. (B) Aliquots of the *in vitro* SUMOylation samples, used for the experiment described in Fig. 3A, were used to verify SUMOylation efficiency by immunoblotting with an antibody against KIF4A.

Does SUMO modification affect the activity of the motor protein KIF4A? We performed an ATPase activity assay to follow KIF4A activity in the absence and presence of modification by SUMO2. Recombinant GST–KIF4A WT or its SUMOylation-deficient mutants (K460R and E458A/E461A) were incubated with SUMOylation mix in the absence or presence of SUMO2 to generate KIF4A proteins either modified or not by SUMO2. ATPase activity assays were performed in the absence or presence of MTs, since MT binding is required for specific KIF4A ATPase activity as required for motor activity (Nunes Bastos et al., 2013). GST–KIF4A WT was active in the presence of MTs as expected, but SUMOylation did not affect the ATPase activity of KIF4A (Fig. 3A). Aliquots of the samples were analyzed by immunoblotting to confirm *in vitro* SUMOylation (Fig. 3B). This suggests that both KIF4A mutants are functional for MT binding and dimerization, since both are essential for ATPase activity, and SUMO modification is not critical for KIF4A motor activity.

### Mutating lysine 460 in endogenous KIF4A via genome editing by CRISPR-Cas9 abolishes SUMOylation

**Fig. 4.**
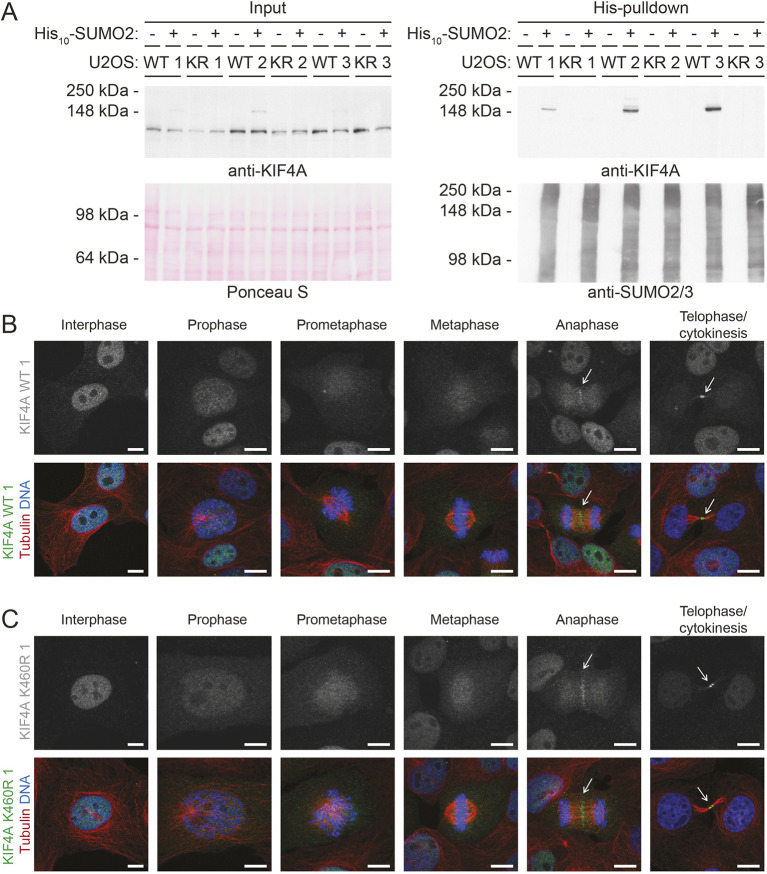
**The localization of endogenous KIF4A during mitosis is not dependent on SUMOylation.** (A) Three KIF4A mutant (K460R) clones and their KIF4A wild-type (WT) control clones obtained by CRISPR-Cas9-directed genome editing were infected with lentivirus encoding His_10_–SUMO2 and selected with puromycin to obtain stable cell lines. Subsequently, these cells were lysed. A His_10_ pulldown was performed to enrich for SUMOylated proteins. Input and pulldown samples were analyzed by immunoblotting using antibodies against KIF4A and SUMO2/3, while Ponceau S staining was used to confirm equal loading. (B,C) U2OS clone 1 with (B) endogenous KIF4A (WT) or (C) with endogenous SUMOylation-deficient KIF4A (K460R) were grown on glass slides, fixed and stained with antibodies against KIF4A (green), tubulin (red) and Hoechst 33258 to visualize DNA (blue). Representative images were taken of cells in subsequent stages of mitosis to visualize KIF4A localization. Arrows highlight midzones and midbodies. Each experiment was performed at least three times. Scale bars: 10 µm.

We next studied the role of KIF4A SUMOylation *in vivo* by genetic manipulation in U2OS cells. CRISPR-Cas9-directed genome editing was used in combination with a repair template to mutate the endogenous *KIF4A* gene to yield the K460R mutant. Each clone was tested by PCR and digestion with restriction enzymes, followed by sequencing of candidates. Finally, three clones were identified for which the corresponding codon AAA encoding lysine 460 was mutated into codon AGA, encoding an arginine (Fig. S3). For each KIF4A mutant (K460R) clone, an individual KIF4A control (WT) clone was selected which had undergone exactly the same procedure at the same moment without introducing any mutations. To check SUMOylation of KIF4A, these three WT and three K460R clones were infected with lentivirus encoding His_10_–SUMO2 and selected by puromycin to obtain stable cell lines. His_10_ pulldown and immunoblot analysis revealed complete abolishment of KIF4A SUMOylation in all three KIF4A K460R mutant clones, while WT KIF4A in all three control clones was confirmed to be SUMOylated (Fig. 4A).

Since SUMOylation can affect the subcellular localization of target proteins, cells of each clone were plated on glass slides and stained for endogenous KIF4A by immunofluorescence. Co-staining against tubulin and DNA enabled recognition of cells in various stages of mitosis. WT KIF4A was observed to localize primarily at the midzone during anaphase and at the midbody during cytokinesis in all three control clones (Fig. 4B; Fig. S4A). No differences in localization were observed for K460R KIF4A in any of the three mutant clones (Fig. 4C; Fig. S4B). Thus, the subcellular localization of KIF4A is not regulated by SUMOylation at position K460.

### SUMOylation of endogenous KIF4A regulates abscission

**Fig. 5.**
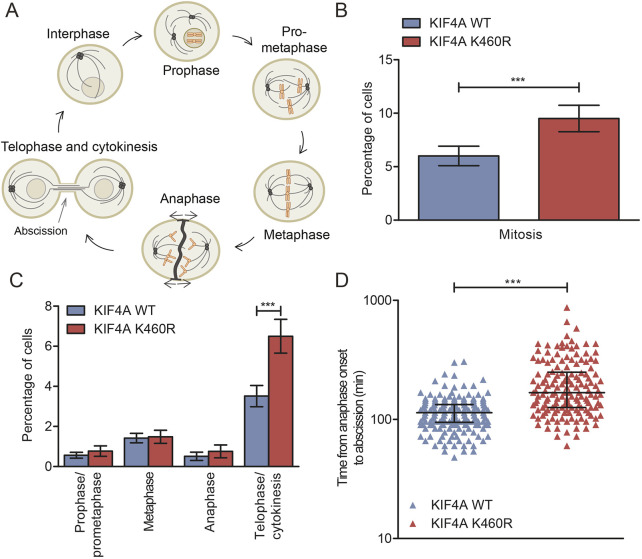
**SUMOylation of endogenous KIF4A regulates abscission.** (A) Cartoon depicting the subsequent phases of mitosis during cell division. (B) Three independent sets consisting of a KIF4A WT and K460R clone were fixed and stained as described in Fig. 4B,C. Random images were taken and the total amount of cells and the number of cells in mitosis per field were analyzed for at least 400 cells per clone per experiment. The percentage of cells in mitosis was averaged for the three WT and the three K460R KIF4A clones. Mean±s.d. values are shown. ***P<0.0005 (two-sided Student's *t*-test). (C) The mitotic cells identified in the experiment described in Fig. 5B were categorized into prophase/prometaphase, metaphase, anaphase or telophase/cytokinesis. The percentage of cells in each phase of mitosis was averaged for the three WT and the three K460R clones. Mean±s.d. values are shown. ***P<0.0005 (two-sided Student's *t*-test). Each experiment was performed at least three times. (D) All three WT and three K460R KIF4A clones were transfected with a GFP–tubulin construct, selected using G418, subcloned and sorted by flow cytometry to obtain stable cell lines with equal GFP–tubulin expression. Live-cell imaging was used to analyze the time from anaphase onset to abscission, based on the DIC and GFP–tubulin signal for 66 cells per WT clone and 51 cells per K460R clone. The data obtained from the individual WT as well as the individual K460R clones were pooled and each cell is represented by a colored triangle. Median values with interquartile ranges are shown in black. ***P<0.0005 (Mann–Whitney test).

While studying the samples to determine KIF4A localization during mitosis (Fig. 5A), the relative amount of cells in mitosis caught our attention. Indeed, upon quantification, an increased percentage of mitotic cells was observed for each K460R mutant clone compared to its WT control clone (Fig. S5A). The results obtained for all three WT cell lines and the results obtained for all three K460R cell lines were averaged (Fig. 5B). To determine whether the increased percentage of cells in mitosis could be attributed to a specific mitotic phase, cells were categorized into prophase/prometaphase, metaphase, anaphase and telophase/cytokinesis. This detailed analysis revealed an increased percentage of cells in telophase/cytokinesis for the K460R clones compared to their corresponding WT clones (Fig. 5C; Fig. S5B), suggesting a role for SUMOylated KIF4A during this final stage of cell division.

To study this phenotype in more detail, the WT and K460R clones were transfected with GFP–tubulin and selected to create stable cell lines (Fig. S5C). Live-cell imaging was performed using differential interference contrast (DIC) to visualize the moment of anaphase onset and GFP–tubulin to identify disconnection of the intercellular MT bridge at the end of cytokinesis, which was marked as the moment of abscission. Mitotic cells with a K460R KIF4A background needed significantly longer (a median of 168 min) to proceed from anaphase onset to abscission compared to cells expressing WT KIF4A protein (a median of 114 min) (Fig. 5D; Fig. S5D). These data indicate that the increased percentage of cells in cytokinesis is the result of an abscission delay, uncovering a SUMO-regulated function for KIF4A in this final step of cell division.

### SUMOylation of KIF4A enhances binding to MT destabilizer STMN1

**Fig. 6.**
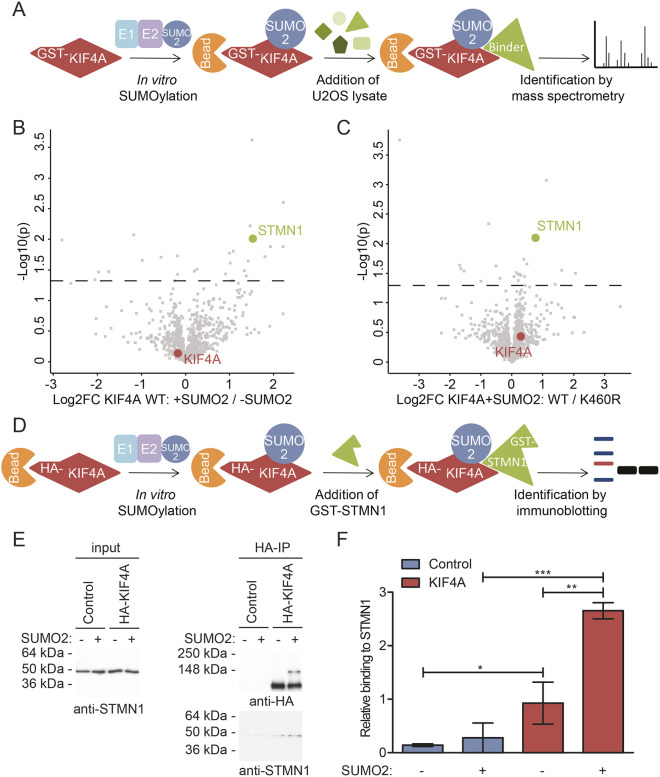
**SUMOylation of KIF4A enhances binding to STMN1.** (A) A cartoon depicting the experimental set-up to identify proteins binding preferentially to SUMOylated KIF4A. Recombinant GST–KIF4A WT or its SUMOylation-deficient mutant (K460R) were bound to beads and *in vitro* SUMOylation assays were performed. In control assays, SUMO2 was omitted. Subsequently, U2OS cell lysates were added, incubation was performed, samples were washed and bound proteins were identified by mass spectrometry. (B) Volcano plot showing proteins binding preferentially to SUMOylated over non-SUMOylated GST–KIF4A. On the *x*-axis, the Log2 fold change (FC) between the proteins identified by mass spectrometry in the samples bound to SUMOylated GST–KIF4A WT versus non-SUMOylated GST–KIF4A WT is indicated. On the *y*-axis, the −Log10(*P*) values for the identified proteins are shown. All proteins above the dashed line were considered significantly different between the two datasets (*P*<0.05). (C) Volcano plot showing proteins binding preferentially to SUMOylated GST–KIF4A WT over the K460R mutant. The Log2FC and −Log10(*P*) values for the identified proteins are shown. Proteins above the dashed line were considered significantly different between the two datasets (*P*<0.05). (D) Cartoon depicting the experimental procedure employed to verify STMN1 as a differential binding protein, as identified by mass spectrometry. U2OS cells were transfected with a control or HA–KIF4A WT construct and lysed after 3 days. Upon purification via HA IP, an *in vitro* SUMOylation assay was performed and in the negative control SUMO2 was omitted. Finally, the samples were incubated with recombinant GST–STMN1, washed extensively and eluted. (E) Input and IP samples from the experiment described above were analyzed by immunoblotting using antibodies against the HA tag and STMN1. (F) Quantification of the result of three independent experiments presented as in Fig. 6E. The mean±s.d. relative binding of GST–STMN1 to KIF4A is shown. *P<0.05, **P<0.005, ***P<0.0005 (two-sided Student's *t*-test). Each experiment was performed at least three times.

SUMOylation of target proteins can affect their binding to other proteins. To determine whether SUMOylation contributes to proper regulation of abscission through affecting binding of KIF4A to specific partners, a mass spectrometry screen was performed (Fig. 6A). Recombinant GST–KIF4A WT and its K460R mutant were incubated with SUMOylation mix (Fig. S6A). As an additional negative control, SUMO2 was omitted as indicated. Samples were bound to beads and washed, prior to incubation with U2OS cell lysate. After additional washing and sample preparation, peptides were measured by mass spectrometry. Furthermore, aliquots of the protein samples were analyzed by immunoblotting to confirm the presence of (SUMOylated) KIF4A after incubation with the U2OS cell lysate (Fig. S6B). Sample analysis revealed a significant preference of the MT destabilizer stathmin 1 (STMN1) for SUMOylated WT KIF4A compared to non-SUMOylated WT KIF4A (Fig. 6B). Differential binding of STMN1 was also found by mass spectrometry when comparing SUMOylated WT KIF4A with K460R KIF4A that was incubated with the complete SUMOylation mix (Fig. 6C).

Preferential binding of recombinant GST–STMN1 to SUMOylated KIF4A was subsequently confirmed by immunoblotting (Fig. 6D,E). Whereas STMN1 also binds to non-SUMOylated KIF4A, quantification of three independent experiments confirmed increased binding of STMN1 to SUMOylated KIF4A (Fig. 6F).

## DISCUSSION

**Fig. 7.**
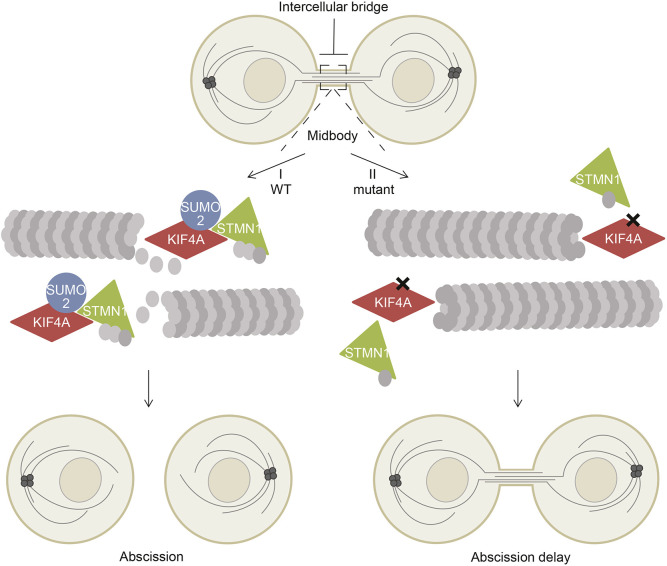
**Model of how SUMOylation of KIF4A enhances binding to MT destabilizer STMN1 and promotes abscission.** Cartoon depicting the intercellular bridge between the newly formed daughter cells during cytokinesis. (I) In cells expressing wild-type KIF4A, SUMOylation of KIF4A enhances its binding to STMN1 at the midbody. Subsequently, MT destabilization by STMN1 is promoted, which results in abscission and thereby completion of the final step of cell division. (II) Blocking KIF4A SUMOylation reduces STMN1 binding. Consequently, this results in reduced MT destabilization by STMN1 at the midbody and ensuing delay in abscission.

The function of SUMO, a small ubiquitin-like modifier, is considerably less understood than that of ubiquitin itself. Here, we studied the role of the SUMO modification of KIF4A. We identify an important role of SUMOylated KIF4A in the regulation of abscission as this controls the interaction with the MT destabilizer STMN1. This is summarized in a model in Fig. 7. SUMOylation of KIF4A increases its affinity for STMN1. Since KIF4A is a plus-end-directed motor protein, enhanced binding of STMN1 to SUMOylated KIF4A could affect the relative concentration of STMN1 along MTs, possibly enhancing its concentration at the plus-end (Nouar et al., 2016). This could lead to local MT destabilization to enable timely separation of daughter cells. Interestingly, no phosphorylated peptides of this MT destabilizing protein were identified by our mass spectrometry, indicating that SUMOylated KIF4A interacts with the active form of STMN1 (Larsson et al., 1997). In the absence of KIF4A SUMOylation, decreased binding of KIF4A to STMN1 may result in slower MT destabilization, and subsequently a delay in cytokinesis. Whereas it was previously found that SUMO2/3, KIF4A and STMN1 are all important players in the earlier stages of mitosis (see Introduction), our new results suggest that these proteins team-up in a dynamic model to regulate cytokinesis, the final step of cell division.

The SUMOylation site and SIM in KIF4A are well conserved among higher eukaryotes, but are missing in model lower eukaryotes such as *Drosophila melanogaster* and *Caenorhabditis elegans* (Fig. S7). This corresponds with the absence of STMN1 in these lower eukaryotes. Interestingly, these conservation patterns correlate exactly with the process of open mitosis, characteristic for higher eukaryotic cells (Güttinger et al., 2009).

Mechanistically, we report the first *in vitro* SUMOylation via the inverted SUMO consensus motif (Matic et al., 2010) upon addition of only SUMO E1 and SUMO E2, indicating that UBC9 is able to directly recognize and modify the ExK motif. The SIM in KIF4A is essential for its SUMOylation and most likely responsible for the recruitment of the SUMO-charged UBC9 complex either or not employing a SUMO conjugated to UBC9 itself (Pichler et al., 2017).

To gain more insight into the effect of SUMO on KIF4A function, we employed CRISPR-Cas9 genome editing and obtained cell lines expressing endogenous SUMOylation-deficient mutant KIF4A. To the best of our knowledge this is the first time this technique was employed to study a SUMO target protein at the endogenous level, which avoids the risks of incorrect and heterogeneous expression levels of exogenous constructs. Moreover, it overcomes the challenge of missing out on potential phenotypes due to partial knockdown. Clearly, CRISPR-Cas9 genome editing should gain momentum to reveal the functional relevance of specific post-translational modification events in the SUMO field and beyond.

By mutating a single lysine in KIF4A, we were able to reveal a specific function of SUMO-modified KIF4A in abscission. Previous research using silencing techniques claimed a role of the multifunctional KIF4A protein earlier in mitosis (Hu et al., 2011; Kurasawa et al., 2004; Zhu and Jiang, 2005). In this case, the function of KIF4A in abscission was missed because this stage of mitosis is not properly reached in the absence of KIF4A. Our study indicates that additional functions might be uncovered for other proteins by focusing on the modification level rather than the total protein level although investigating proteins regulated by post-translational modification at this level is challenging. Interestingly, our results show that SUMO is able to regulate key cellular functions by the modification of single target proteins in addition to its role in protein group modification (Psakhye and Jentsch, 2012). As a result of co-regulation of functional groups by SUMOylation, phenotypes of single target proteins deficient for SUMOylation are generally modest and quite often, no phenotypes can be found at all.

SUMOylation levels of KIF4A appeared to be rather stable during cell cycle progression, indicating that KIF4A is continuously modified and demodified at constant rates throughout the cell cycle. This is a fairly puzzling finding, but also intriguing and novel, showing that a stable modification has a function at a specific phase of mitosis (i.e. abscission). This could be due to limited opportunities for the enzymatic machinery to carry out the modification during cytokinesis. One thought here is that the SUMO-conjugating machinery is equipped with DNA-binding domains, limiting SUMO conjugation primarily to the presence of substrates at chromatin. Therefore, the modification has to happen prior to cytokinesis, despite its function in abscission.

In summary, we have uncovered a novel role for SUMOylated KIF4A in cytokinesis, where it plays a role in abscission. The process of cytokinesis is tightly regulated to ensure daughter cells are not separated until sister chromatid segregation is completed. We now place SUMO modification of KIF4A and the resulting association with STMN1 in a dynamic model that is critical in the last phase of mitosis, as shown in Fig. 7. How dynamic modification by SUMO of KIF4A is controlled during mitosis is unclear. But a complex SUMO-controlled event determines the final step in mitosis.

## MATERIALS AND METHODS

### CRISPR-Cas9-directed genome editing

To obtain cell lines in which the triplet encoding lysine 460 in the endogenous KIF4A was replaced by a triplet encoding arginine, CRISPR-Cas9-directed genome editing was used in combination with a repair template enabling targeted mutagenesis. The pSpCas9(BB)-2A-GFP plasmid (Ran et al., 2013) was digested with BbsI (New England Biolabs) and purified from a 1% TAE agarose gel using the Wizard^®^ SV Gel and PCR Clean-up System (Promega). Two guide RNA primers were designed containing BbsI overhangs and the target sequence upstream of the selected protospacer adjacent motif (PAM) region close to the sequence encoding lysine 460 [forward (FW), 5′-CACCGAGTGGAGACTTTGGAAGACC-3′; and reverse (RV), 5′-AAACGGTCTTCCAAAGTCTCCACTC-3′]. These primers were annealed and phosphorylated by T4 polynucleotide kinase (New England Biolabs), before ligation into the BbsI digested plasmid by T4 ligase (New England Biolabs). A repair template was designed to contain the desired lysine to arginine mutation and several silent mutations to prevent recognition of the repaired gene by the guide RNA and to introduce a recognition site for the restriction enzyme TaqαI (5′-GTCATATGAATAACCCTTGGGTTCTTTTTGTTTGAATTTTCAGCTGCAAACTGGATCTTCAAAAGCTTGTCGAGACATTGGAGGATCAGGAATTGAGAGAAAATGTAGAGATAATTTGTAACCTGCAGCAATTGATTACCCAGTTATCGGTAAGCCAAGTAGGGGCAGTGTAAAT-3′), and obtained as 4 nmole ultramer (Integrated DNA Technologies). Two million U2OS cells were transfected with 3 µg of Cas9/GFP/guideRNA encoding plasmid and 6 µl of 10 µM repair template using 14.4 µl Lipofectamine 2000 (Invitrogen) in Optimem (Life Technologies). At 3 days after transfection, cells were sorted by flow cytometry and GFP-positive cells were plated at 5000 cells per 15 cm dish. Colonies were picked and maintained in 24-well plates. DNA was purified from each clone and part of the *KIF4A* gene was amplified by PCR using GoTaq polymerase (Promega) and the following primers: FW, 5′-ACCGCCACTGATGATCTCCTG-3′ and RV, 5′-ATCAAATGATCCACGACCTCTGTCC-3′. The PCR product was digested by TaqαI (New England Biolabs), to determine which cells had used the designed template to repair the break caused by Cas9. Candidate clones and several control clones were sequenced to confirm their identity. This procedure was repeated until three correctly mutated clones were identified. Each mutant clone was matched to a clone that had undergone exactly the same procedure but was identified with a sequence encoding wild-type (WT) KIF4A, resulting in three individual sets of a WT and a K460R clone.

### Cell culture and cell lines

Unless otherwise stated, human embryonic kidney 293 (HEK293T) cells (DuBridge et al., 1987) and human bone osteosarcoma epithelial (U2OS) cells (ATCC) were cultured at 37°C and 5% CO_2_ in Dulbecco's modified Eagle's medium (DMEM, Thermo Fisher Scientific) supplemented with 10% fetal bovine serum (FBS, Thermo Fisher Scientific), 100 U/ml penicillin and 100 µg/ml streptomycin (P/S, Thermo Fisher Scientific). Cells were authenticated and regularly tested for mycoplasma infection and found to be negative. The U2OS cell line stably expressing low levels of His_10_-tagged SUMO2 was obtained by infection with a lentivirus encoding a His_10_–SUMO2 IRES GFP construct and subsequent selection by low GFP expression as described previously (Hendriks et al., 2014). The mature protein that is referred to as SUMO2 has the following amino acid sequence: MSEEKPKEGVKTENDHINLKVAGQDGSVVQFKIKRHTPLSKLMKAYCERQGLSMRQIRFRFDGQPINETDTPAQLEMEDEDTIDVFQQQTGG.

To enable enrichment of SUMOylated endogenous proteins from the three sets of KIF4A WT and K460R clones, the cell lines were infected with lentivirus encoding His_10_–SUMO2 and puromycin resistance (Hendriks and Vertegaal, 2016b) at a multiplicity of infection (MOI) of 3 using 8 µg/ml polybrene. Stable His_10_–SUMO2-expressing cell lines were created by selection with 2.5 µM puromycin (Calbiochem). To enable visualization of MTs by live-cell imaging, the three sets of KIF4A WT and K460R clones were transfected with the pEGFP fused to α-tubulin plasmid (Clontech). After selection by Geneticin (G418, Life Technologies), cells were seeded at 5000 cells per 15 cm dish. GFP-positive colonies were picked based on equal expression levels and additionally sorted by flow cytometry.

### Site-directed mutagenesis

Mutations were introduced in the WT KIF4A construct encoding the canonical KIF4A protein (Uniprot identifier O95239-1) using QuikChange site-directed mutagenesis (Stratagene). The forward and reverse primers used were: K460R, FW, 5′-GACCAGGAATTGAGAGAAAATGTAGAG-3′, RV, 5′-CTCTACATTTTCTCTCAATTCCTGGTC-3′; E458A, FW, 5′-GTGGAGACTTTGGAAGACCAGGCGTTGAAAGAAAATGTAGAG-3′, RV 5′-CTCTACATTTTCTTTCAACGCCTGGTCTTCCAAAGTCTCCAC-3′; E461A, FW, 5′-GGAAGACCAGGAATTGAAAGCGAATGTAGAGATAATTTG-3′, RV, 5′-CAAATTATCTCTACATTCGCTTTCAATTCCTGGTCTTCC-3′; E458A/E461A, FW, 5′-CTTTGGAAGACCAGGCGTTGAAAGCGAATGTAGAG-3′, RV, 5′-CTCTACATTCGCTTTCAACGCCTGGTCTTCCAAAG-3′; ΔSIM FW, 5-CTGAAAGTGTCTTACTTAGAGATTTACAATGAAGAAGCTGCGGATGCTGCATGCCCATCTCGTGAGAAAGC-3′, RV 5′-GCTTTCTCACGAGATGGGCATGCAGCATCCGCAGCTTCTTCATTGTAAATCTCTAAGTAAGACACTTTCAG-3′.

### Transfection and lentivirus production

Cells were plated in FBS-containing DMEM without P/S. To overexpress the indicated protein, U2OS cells were transfected with 24 µg plasmid DNA and 60 µg polyethylaneimine (PEI) at 60% confluency in a 15 cm dish. Medium was replaced for DMEM containing FBS and P/S after 24 h, and cells were washed with PBS and lysed 3 days after transfection to obtain total lysate samples (input) and samples for protein purification (His_10_ pulldown or HA immunoprecipitation) unless otherwise stated. To produce lentivirus, HEK293T cells were transfected with 7.5 µg pCMV-VSVG, 11.4 µg pMDLg-RRE, 5.4 µg pRSV-REV and 13.7 µg of a plasmid encoding SUMO2 as well as puromycin resistance. The medium was collected 48 and 72 h after transfection and filtered using a 0.45 µm syringe filter (Pall Life Science). The virus titer was determined using p24 ELISA and the sample was stored at −20°C until use.

### His_10_ pulldown

Enrichment of SUMOylated proteins was performed by His_10_ pulldown as described previously (Hendriks et al., 2014). In short, cells were resuspended in lysis buffer, sonicated and equalized using BCA Protein Assay Reagent (Thermo Fisher Scientific). Lysates were incubated with Ni-NTA beads (Qiagen) overnight at 4°C, washed and eluted for 30 min at room temperature (RT).

### HA immunoprecipitation

To detect modification of exogenous HA-tagged KIF4A with endogenous SUMO2/3, an HA immunoprecipitation (IP) was performed to preserve SUMOylation on target proteins according to the following protocol. Cells were lysed in buffer 1, containing 1% SDS, 0.5% NP-40, 50 mM NaF, 1 mM NaVO_3_, 5 mM β-glycerol phosphate, 5 mM sodium pyruvate (NaPy), 0.5 mM EGTA, 5 mM PNT, protease inhibitors with EDTA, 70 mM chloroacetamide (CAA) and 1× PBS. Samples were equalized using BCA Protein Assay Reagent and diluted with an equal volume of buffer 2 (2% Triton X-100, 0.5% sodium deoxycholate, 1% BSA, 5 mM NaF, 1 mM NaVO_3_, 5 mM β-glycerol phosphate, 5 mM NaPy, 0.5 mM EGTA, 5 mM PNT, protease inhibitors with EDTA, 70 mM CAA and 1× PBS). Upon centrifugation at 4°C and 13,200 rpm (15,000 ***g***) for 45 min, the supernatant was collected and incubated with EZview red anti-HA affinity gel (Sigma) for 90 min at 4°C while rotating. Samples were washed four times with buffer 3, containing 50 mM Tris-HCl pH 7.5, 150 mM NaCl, 0.5% NP-40, 5 mM NaF, 1 mM NaVO_3_, 5 mM β-glycerol phosphate, 5 mM NaPy, 0.5 mM EGTA, 5 mM PNT, protease inhibitors with EDTA and 70 mM CAA. Next, samples were washed twice with buffer 4, containing 50 mM Tris-HCl pH 7.5, 150 mM NaCl and 0.5% NP-40. Finally, samples were eluted for 30 min at RT and 1200 rpm (500 ***g***) in buffer 4 supplemented with 100 µg/ml HA-peptide (Sigma).

To purify HA-tagged KIF4A for *in vitro* SUMOylation reactions, an HA IP was performed in a non-denaturing manner according to the following protocol. Cells were lysed in buffer 5, containing 30 mM HEPES pH 7.6, 130 mM NaCl, 1 mM MgCl_2_, 0.5% Triton X-100, protease inhibitors with EDTA and 70 mM CAA. Samples were incubated on ice for 10 min, before centrifugation at 4°C and 15,000 ***g*** for 10 min. The supernatant was collected and equalized using BCA Protein Assay Reagent. After incubation with EZview red anti-HA affinity gel for 90 min at 4°C while rotating, samples were washed six times in buffer 5. Finally, samples were eluted in buffer 5 supplemented with 100 µg/ml HA-peptide at RT and 1200 rpm (500 ***g***) for 30 min.

### SUMOylation of eluted HA–KIF4A

HA–KIF4A enriched from 4×10^6^ U2OS cells by HA-IP as described above was *in vitro* SUMOylated. Unless otherwise stated, samples were incubated in a total volume of 20 µl for 3 h at 4°C with SUMOylation mix, containing 50 mM Tris-HCl pH 7.5, 5 mM MgCl_2_, 3.5 U/ml creatine kinase, 10 mM creatine phosphate, 1.1 U/ml inorganic pyrophosphatase, 2 mM ATP, 600 ng SUMO E1, 2 µg SUMO E2 and 4.4 µg SUMO2 Kzero (K0) (made in-house). SUMO2 K0 refers to a mutant SUMO2 in which all lysine residues were mutated into arginine residues to prevent SUMO chain formation.

### Electrophoresis, immunoblotting and antibodies

For each experiment, the collected cells were divided in at least two aliquots, including one for total lysate samples (input) and one for protein purification (His_10_ pulldown or HA IP). Inputs were lysed in input buffer (1% SDS, 1% NP-40, 50 mM Tris-HCl pH 7.5 and 150 mM NaCl), incubated at 99°C for 10 min at 1200 rpm (500 ***g***) and equalized using BCA Protein Assay Reagent, followed by immunoblot analysis. Purification samples were prepared according to the His_10_ pulldown or HA IP protocol. For immunoblot analysis, dithiothreitol (DTT, Sigma) and NuPAGE LDS Sample Buffer (LDS, Life Technologies) were added, and each sample was incubated at 70°C for 10 min. Proteins were separated by gel electrophoresis on 8% or 15% gels made in-house in Tris-glycine buffer for 75 min at 150 V. Proteins were transferred to Hybond nitrocellulose membranes (GE Healthcare) in cold transfer buffer at 25 V for 3 h. Subsequently, proteins were stained with Ponceau S (Sigma) to confirm equal loading and membranes were blocked in PBS with 0.05% Tween-20 (Merck) (PBS/T) and 8% milk powder (blocking solution) for 1 h at RT. Membranes were incubated overnight at 4°C with primary antibodies diluted in blocking solution, including rabbit polyclonal anti-KIF4A (Bethyl, A301-074A, 1:2000), mouse monoclonal anti-SUMO2/3 (Abcam, 8A2, 1:2000), mouse monoclonal anti-HA.11 (Sanbio, MMS-101R, 1:1000), mouse monoclonal anti-α-tubulin (Sigma, T6199, 1:1000), rabbit polyclonal anti-GFP (Novus Biologicals, NB600-308, 1:1000) and rabbit monoclonal anti-STMN1 (Cell Signaling Technology, D1Y5A, 1:1000). Membranes were washed three times for 10 min at RT in PBS/T, before incubation with HRP-coupled secondary donkey anti-rabbit-IgG or goat anti-mouse-IgG antibodies (Pierce) diluted in blocking solution for 1 h at 4°C. Upon washing in PBS/T three times for 10 min at 4°C, Pierce ECL 2 immunoblotting substrate (Life Technologies) was used to visualize the signal on RX Medical films (Fuji).

### Cell synchronization and fluorescence-activated cell sorting analysis

Two blocking agents were used to synchronize cells into specific cell cycle stages. Thymidine (Sigma) was added at a concentration of 4 mM to block cells at the border of G1 to S phase. After 16 h, cells were lysed immediately (G1 phase) or washed twice with PBS to release them from their cell cycle arrest. These cells were either released into DMEM with FBS and P/S for 5 h (early S phase) or 8 h (late S phase) or into DMEM with FBS, P/S and 0.1 µg/ml nocodazole (Sigma) to block cells in prometaphase. After 20 h, these arrested cells were lysed immediately (early M phase) or released into DMEM with FBS and P/S for 30 min (mid M phase), 2 h (late M phase), 4 h (G1 phase) or 8 h (G1/S phase). Cells were collected using trypsin treatment and washed with PBS. Finally, each sample was divided into three aliquots, which were fixed for fluorescence-activated cell sorting (FACS) analysis, lysed to perform a His_10_ pulldown or lysed to obtain input samples.

The samples harvested for FACS analysis were fixed in 70% ice-cold ethanol and incubated at 4°C for at least 16 h. Samples were prepared on the day of measurement by flow cytometry, starting with centrifugation at 1200 rpm (500 ***g***) for 2 min and washing in PBS with 2% FBS. Upon a second centrifugation step, cells were resuspended in PBS containing 2% FBS, 25 µg/ml propidium iodide (Sigma) and 100 µg/ml RNase A (Sigma). Samples were stained for 30 min at 37°C, followed by measuring the cellular DNA content by flow cytometry with the BD LSRII system and BD FACS DIVA software (BD Bioscience Clontech).

### ATPase activity assay

Recombinant GST–KIF4A wild-type (WT) or SUMO deficient mutants (K460R and E458A/E461A) were *in vitro* SUMOylated by incubation at 4°C for 3 h. The complete SUMOylation mix used for one ATPase activity reaction contained 2.5 µg GST–KIF4A, 750 ng SUMO E1, 2.5 µg SUMO E2 and 5.5 µg SUMO2 K0 in 50 mM Tris-HCl pH 7.5, 5 mM MgCl_2_ and 2 mM ATP (dissolved in PIPES). As a non-SUMOylated control, the same reaction was performed but omitting SUMO2 K0. Aliquots of all SUMOylation reactions were saved for immunoblot analysis. To measure ATPase activity the Kinesin ATPase end-point assay (Cytoskeleton) was used according to the manufacturer's instructions, except for the use of 300 µl wells and therefore doubling all reaction volumes. As an additional control, all experimental conditions were also incubated in the absence of MTs to determine background activity. Absorbance was measured at 650 nm with a Synergy HT spectrophotometer (BioTek). Three independent experiments were performed and average relative absorbance with standard deviations were calculated. A two-sided Student's *t*-test was used to determine *P*-values.

### Immunofluorescence

All three sets of WT and K460R KIF4A clones were plated on glass slides in 24-well plates. After 2 days, cells were fixed for 15 min at RT in 4% PFA and washed four times in PBS. Cells were permeabilized with 1% Triton X-100 in PBS for 15 min at RT, followed by washing twice with PBS and once with PBS/T. Samples were blocked for 10 min in TNB, containing 100 mM Tris-HCl pH 7.5, 150 mM NaCl and 0.5% blocking reagent (Roche). After incubation for 1 h at RT with primary antibodies against KIF4A and tubulin diluted in TNB, coverslips were washed five times with PBS/T. Then, cells were incubated for 1 h at RT with secondary antibodies (Alexa-Fluor-488-conjugated goat anti-mouse-IgG and Alexa-Fluor-594 goat anti-rabbit-IgG) diluted in TNB and washed five time with PBS/T. Coverslips were dehydrated by incubating for 1 min each with 70%, 90% and 100% ethanol. Finally, coverslips were mounted onto a microscopy slide using ProLong Gold (Life Technologies) containing 5 µg/ml Hoechst 33258 (Life Technologies).

For high-resolution images, mitotic cells were visualized using a Leica TCS SP8 confocal microscope with a 63× objective. *Z*-stacks were made of cells from top to bottom with 0.4 µm steps at a 1024×1024 format with a speed of 100 Hz. Finally, maximum projections were made and shown in the figures. To determine the percentage of mitotic cells, random images were taken on a Leica DM6B fluorescence microscope. The numbers of total cells and of mitotic cells were analyzed per image for at least 400 cells per clone per experiment. The percentage of cells in mitosis and in each subphase was calculated for six independent experiments and averaged for each clone. The means with standard deviations are shown in the supplementary figures. The results for the three WT and three K460R cell lines were averaged, for which the data with standard deviations are shown in the main figures. A two-sided Student's *t*-test was used to calculate *P*-values.

### Live-cell imaging

Cells were grown on glass-bottomed CG imaging chambers (Zell-Kontakt) in DMEM supplemented with FBS and P/S at 37°C in a humidified atmosphere containing 5% CO_2_. Before imaging, the medium was changed to Phenol Red-free CO_2_-independent Leibovitz's L-15 medium (Thermo Fisher Scientific) supplemented with FBS, and the lid was exchanged for a DIC lid (Zell-Kontakt). Movies were acquired using the Eclipse Ti wide-field microscope (Nikon) with a Plan Apo λ 60× NA 1.4 objective at 37°C in an environment chamber. Five optical sections were collected every 1.5 µm using the ORCA-Flash 4.0 CMOS camera C11440-22CU (Hamamatsu); 2×2 binning was applied for increased signal intensity.

To determine timing from anaphase onset to abscission, mitotic progression of 66 cells per WT clone and of 51 cells per K460R clone was analyzed using Image J software version 1.50e. Anaphase onset was determined as the first frame in which the start of chromosome segregation was visible by DIC. These cells were followed while continuing mitosis until the intercellular MT bridge was disconnected at the end of cytokinesis, and this frame was marked as the moment of abscission. The timing from anaphase onset to abscission for each individual cell is represented by a colored triangle, while the medians with interquartile ranges are shown in black. The results for the individual clones are shown in the supplementary figure, while the main figure shows all 198 WT and 153 K460R cells analyzed from the individual clones pooled together. A Mann–Whitney test was used to calculate *P*-values.

### Identification of GST–KIF4A-binding proteins by mass spectrometry and data analysis

An *in vitro* SUMOylation reaction was performed for 3 h at 4°C with 10 µg recombinant WT or K460R GST–KIF4A in buffer containing 50 mM Tris-HCl pH 7.5, 5 mM MgCl_2_, 2 mM ATP, 3 µg SUMO E1, 10 µg SUMO E2 and 20 µg SUMO K0. The same buffer conditions, except for the absence of SUMO K0, were used as an additional negative control. Samples were incubated with glutathione–Sepharose 4 Fast Flow beads (GE Healthcare) for 1 h at 4°C, followed by washing twice with buffer 6 (50 mM Tris-HCl pH 7.5, and 150 mM NaCl). Subsequently, samples were incubated for 2 h at 4°C with U2OS cell lysate. Lysates were prepared in buffer containing 50 mM Tris-HCl pH 7.5, 150 mM NaCl, 0.5% Triton X-100 and 10 mM N-ethylmaleimide (NEM, Sigma) and protease inhibitors with EDTA. After washing three times with buffer 6, samples were washed three times with 50 mM ammonium bicarbonate (ABC, Sigma) and eluted for 30 min at RT and 1200 rpm (500 ***g***) in ABC complemented with 20 mM glutathione (Sigma).

Samples were passed through pre-washed 0.45 µm filter columns to remove the beads and digested by incubation with 2 µg trypsin (Promega) overnight at 37°C while shaking at 500 rpm. To acidify the samples, trifluoroacetic acid (TFA, Sigma) was added to a final concentration of 2%. Stage tips containing C18 (Sigma) were activated by passing HPLC-grade methanol (Sigma), washed with 80% acetonitrile (ACN, Sigma) in 0.1% formic acid (FA, Sigma) and equilibrated with 0.1% FA. Samples were loaded on these stage tips, washed twice with 0.1% FA and eluted twice with 80% ACN. Finally, samples were vacuum dried using a SpeedVac RC10.10 (Jouan), redissolved in 0.1% FA and transferred to autoloader vials before measurement by mass spectrometry. Four independent experiments were performed and all samples were measured by nanoflow liquid chromatography-tandem mass spectrometry (nanoLC-MS/MS) on an EASY-nLC 1000 system (Proxeon) connected to an Orbitrap Q-Exactive (Thermo Fisher Scientific) through a nano-electrospray ion source. Raw data analysis was performed using Max Quant Software version 1.5.3.30 with its integrated search engine Andromeda. The search was performed against the *in silico* digested proteome containing 92,180 entries of *Homo sapiens* from UniProt (24th March 2016). Label-free quantification was performed using LFQ settings with fast LFQ disabled to quantify all identified peptides (Tables S1 and S2). Proteins identified by the same set of peptides were combined to a single protein group by Max Quant (Tables S1 and S3). These protein groups were further analyzed using Perseus Software version 1.5.2.4. Proteins identified in the categories ‘potential contaminant’, ‘reverse’ or ‘only identified by site’ were removed. The LFQ intensities were log2 transformed and the experimental replicates for each condition were assigned together in an experimental group. Subsequently, all proteins that were not identified in each experimental replicate of at least one experimental group were removed. Missing values were imputed based on the total matrix, using normally distributed values with a randomized 0.3 width (log2) and a 1.8 down shift (log2). Two-sided Student's *t*-tests were performed between the indicated samples to obtain *P*-values and differences for each protein. These values were visualized in a volcano plot, showing *P*-values [as −Log10(*P*)] on the *y*-axis and differences [as Log2FC (fold change)] on the *x*-axis. All proteins with a *P*<0.05 were considered to bind significantly differently between the two indicated experimental groups (Tables S1 and S4).

### GST–STMN1 binding to HA–KIF4A

At 3 days after transfection of U2OS cells with control or HA–KIF4A WT plasmid, cells were lysed in buffer 7 (50 mM Tris-HCl pH 7.5, 5 mM MgCl_2_, 150 mM NaCl and 0.5% Triton X-100). Samples were sonicated and centrifuged at 15,000 ***g*** for 30 min at 4°C. Upon collection of the supernatant, BCA Protein Assay Reagent was used to equalize the samples. Then, samples were incubated with EZview red anti-HA affinity gel for 90 min at 4°C and washed twice in buffer 7. Upon washing twice in buffer 8 (50 mM Tris-HCl pH 7.5 and 5 mM MgCl_2_), an *in vitro* SUMOylation reaction was performed on the beads containing HA–KIF4A enriched from 10^6^ U2OS cells for 3 h at 4°C with rotation in a total reaction volume of 50 µl containing 50 mM Tris-HCl pH 7.5, 5 mM MgCl_2_, 4 mM ATP, 1.5 µg SUMO E1, 5 µg SUMO E2 and 10 µg SUMO K0. Samples were washed three times with buffer 6, followed by incubation with 1 µg recombinant GST–STMN1 WT for 2 h at 4°C with rotation. Finally, samples were washed in buffer 7 and eluted in buffer 7 supplemented with 100 µg/ml HA-peptide at RT and 1200 rpm (500 ***g***) for 30 min. After performing electrophoresis and immunoblotting as described above, the obtained signal was quantified using Fiji version 2.0.0. Three independent experiments were performed and average relative binding with standard deviations were calculated. A two-sided Student's *t*-test was used to determine *P*-values.

## Supplementary Material

Supplementary information
