# Supplementary material for: Chromokinesin KIF4A teams up with stathmin 1 to regulate abscission in a SUMO-dependent manner
Source: J Cell Sci. 2020 Jul 24;133(14):jcs248591. doi: 10.1242/jcs.248591 (PMC7390632; doi:10.1242/jcs.248591)
Supplement: Supplementary information [file joces-133-248591-s1.pdf]

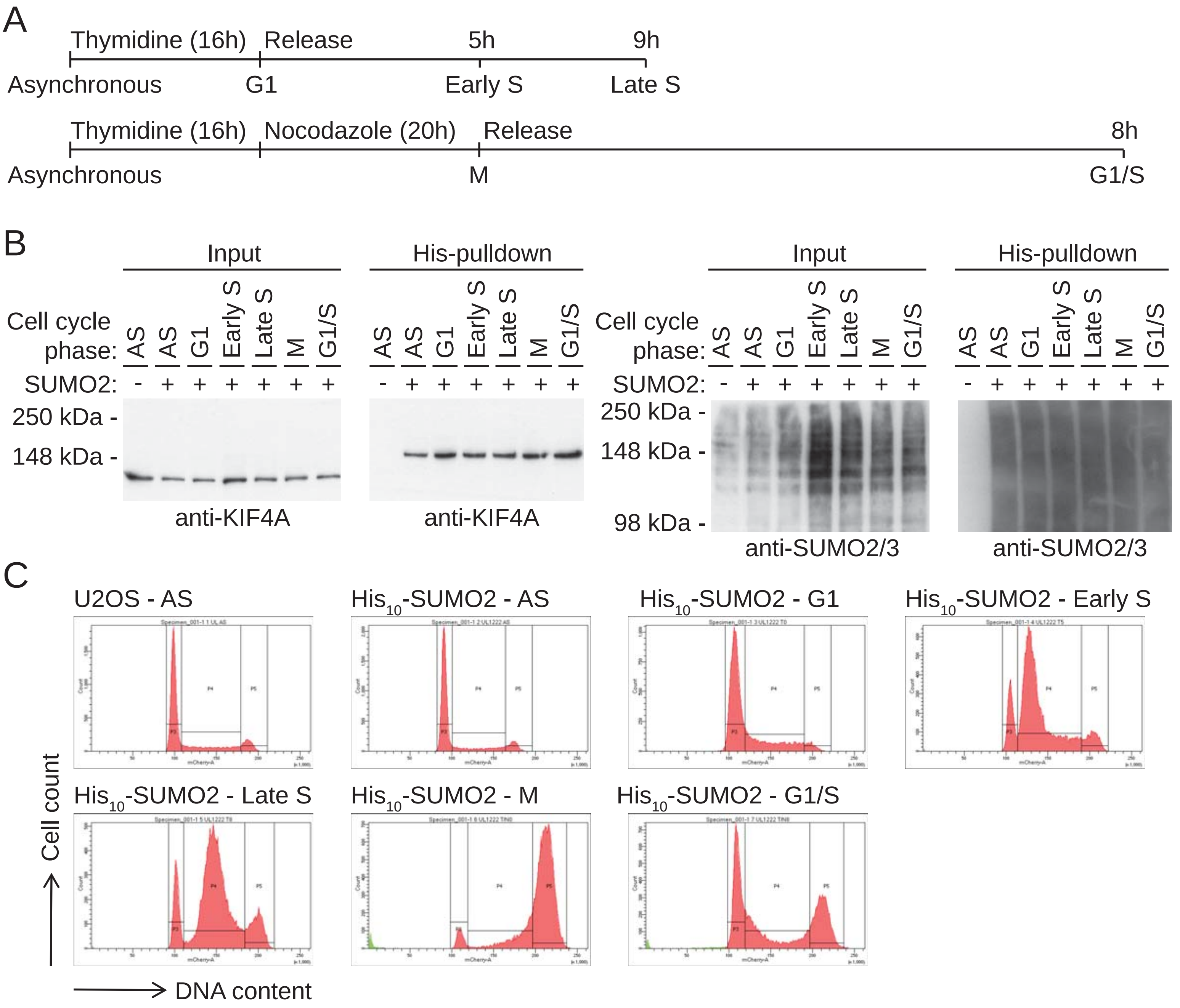

Figure S1 SUMOylation of endogenous KIF4A is stable during cell cycle progression. (**A**) Overview of the cell cycle synchronization strategy indicates the treatment, time of incubation and time of release to obtain cells in the indicated cell cycle phase. (**B**) U2OS cells without or with stable expression of his$_{10}$-SUMO2 were synchronized at the indicated cell cycle phases using thymidine and nocodazole. Cells were lysed and his$_{10}$-pulldowns were performed to enrich for SUMOylated proteins. Samples were analyzed by immunoblotting using antibodies against KIF4A and SUMO2/3. (**C**) Cell cycle synchronization described in Supplementary information, Figure 1B was confirmed by flow cytometry. Each experiment was performed at least three times.

Journal of Cell Science • Supplementary information

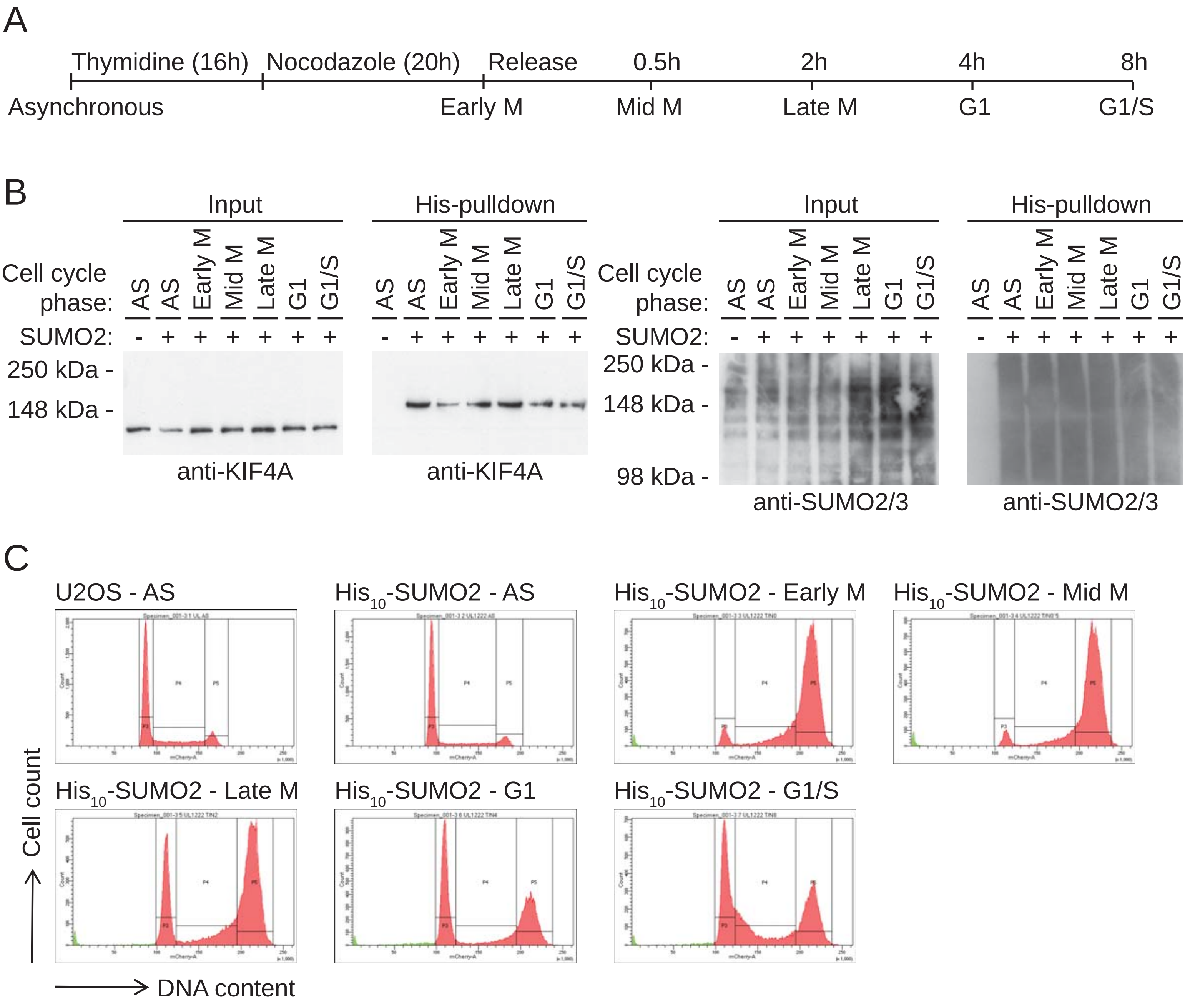

Figure S2 SUMOylation of endogenous KIF4A is stable during mitotic progression. (**A**) Overview of the cell cycle synchronization strategy indicates the treatment, time of incubation and time of release to obtain cells in the indicated mitotic phase. (**B**) U2OS cells without or with stable expression of his$_{10}$-SUMO2 were synchronized in mitosis as indicated using thymidine and nocodazole. Cells were lysed and his$_{10}$-pulldowns were performed to enrich for SUMOylated proteins. Input and pulldown samples were analyzed by immunoblotting using antibodies against KIF4A and SUMO2/3. (**C**) Mitotic cell cycle synchronization described in Supplementary information, Figure 2B was confirmed by flow cytometry. Each experiment was performed at least three times.

Journal of Cell Science • Supplementary information

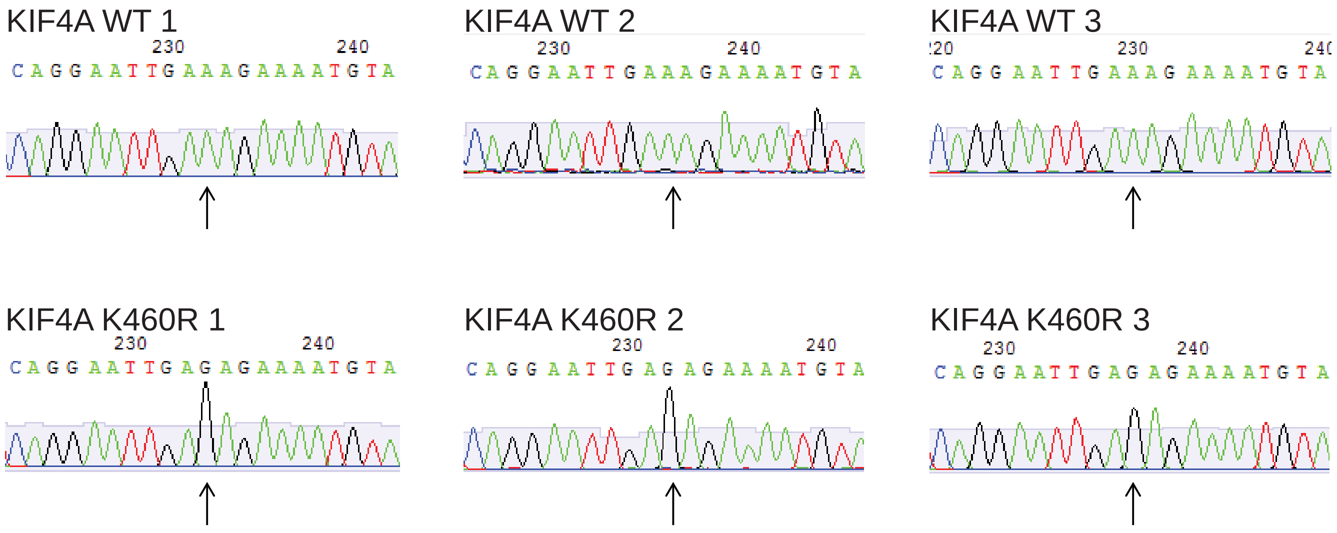

Figure S3 CRISPR-Cas9-directed genome editing was used in combination with a repair template for targeted mutation of the KIF4A gene, resulting in a replacement of the endogenous lysine 460 with an arginine in the KIF4A protein. After subcloning, genome editing was verified by PCR amplification and diagnostic digestion. Subsequently, sequencing was performed to confirm either wildtype (WT) or mutant (K460R) KIF4A identity.

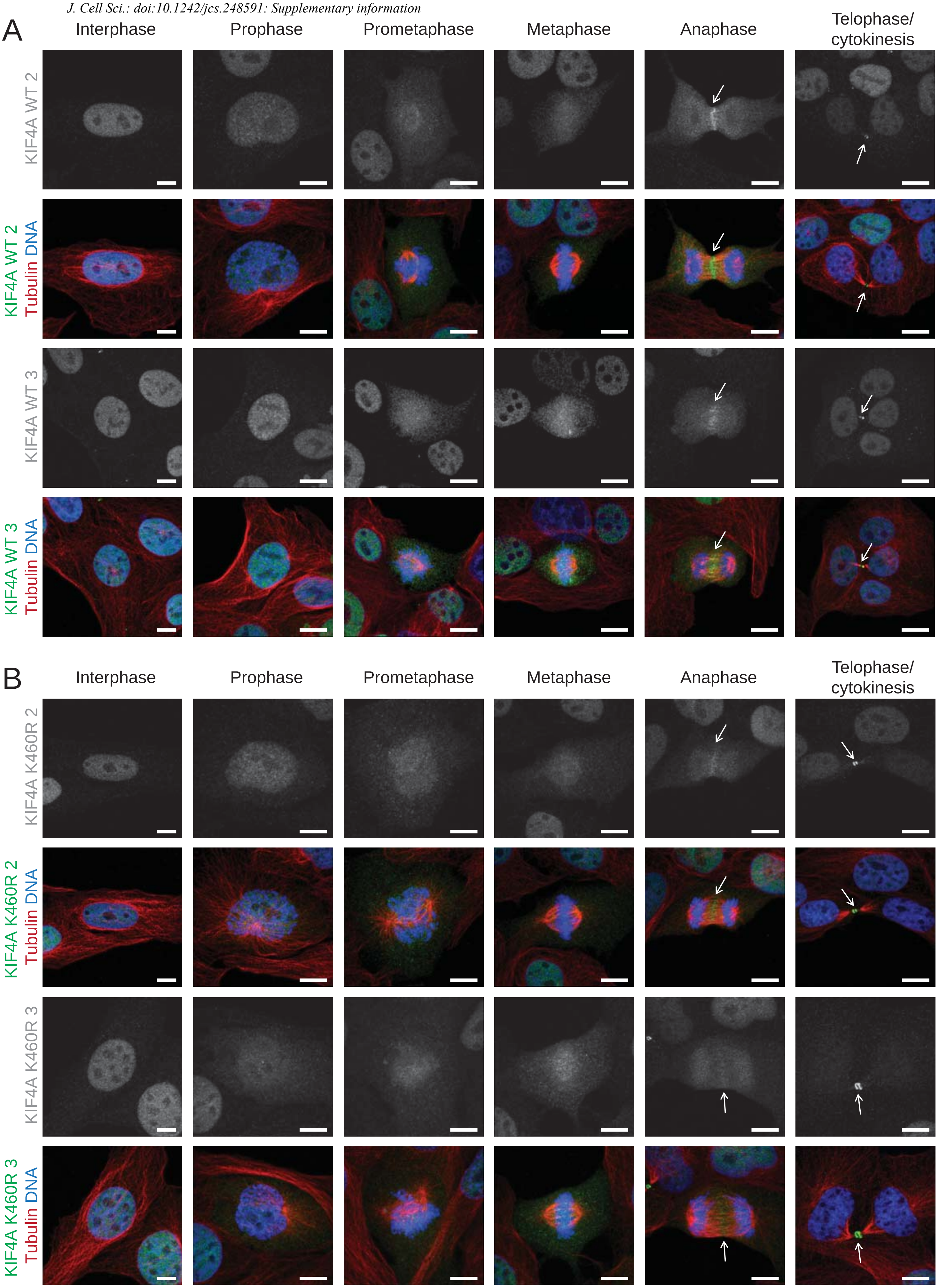

Journal of Cell Science • Supplementary information

Figure S4 The localization of endogenous KIF4A during mitosis is not dependent on SUMOylation. (**A**) Two additional U2OS cell lines expressing endogenous WT KIF4A (clone 2 and 3) or (**B**) endogenous K460R KIF4A (clone 2 and 3) were grown on glass slides, fixed and stained with antibodies against KIF4A (green), tubulin (red) and Hoechst to visualize DNA (blue). Representative images were taken of cells in different stages of mitosis to visualize KIF4A localization. Scale bars correspond to 10 μm. Each experiment was performed at least three times.

Journal of Cell Science • Supplementary information

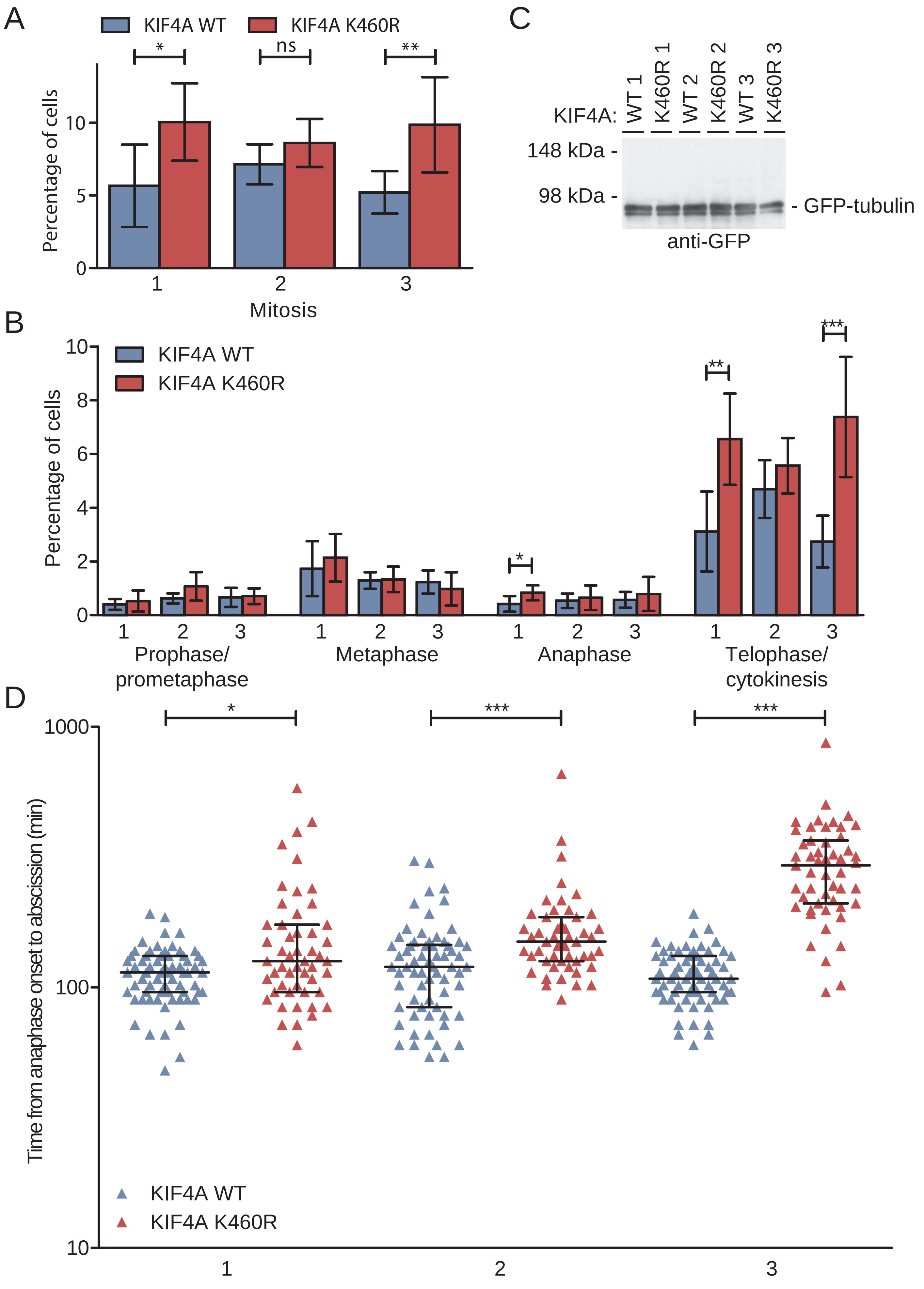
A
KIF4A WT
KIF4A K460R
*
ns
**
Percentage of cells
0
5
10
1
2
3
Mitosis
C
KIF4A:
WT 1
K460R 1
WT 2
K460R 2
WT 3
K460R 3
148 kDa -
98 kDa -
- GFP-tubulin
anti-GFP
B
KIF4A WT
KIF4A K460R
Percentage of cells
0
2
4
6
8
10
*
**
***
1
2
3
Prophase/
prometaphase
Metaphase
Anaphase
Telophase/
cytokinesis
D
Time from anaphase onset to abscission (min)
10
100
1000
*
***
KIF4A WT
KIF4A K460R

Figure S5 Increased mitotic index and delay in abscission in cells deficient for SUMOylation of endogenous KIF4A. (**A**) Three independent sets (1, 2 and 3) of KIF4A wildtype (WT) and SUMOylation deficient (K460R) clones were fixed and stained with Hoechst and antibodies directed against KIF4A and tubulin. Images were taken randomly and analyzed for the total amount of cells and the number of cells in mitosis. The mean percentage of cells in mitosis for each set of KIF4A WT and K460R clones is shown with corresponding standard deviations. P values were calculated using a two-sided Student T-test. One star represents $p < 0.05$ and two stars $p < 0.005$. ns, not significant. (**B**) The mitotic cells identified in the experiment described in Supplementary information, Figure 5A were categorized into prophase/prometaphase, metaphase, anaphase or telophase/cytokinesis. The mean percentage of cells in each phase of mitosis for each set of KIF4A WT and K460R clones is shown with corresponding standard deviations. P values were calculated using a two-sided Student T-test. One star represents $p < 0.05$, two stars $p < 0.005$ and three stars $p < 0.0005$. Each experiment was performed at least three times. (**C**) All three WT and three K460R KIF4A clones were transfected with a GFP-tubulin construct, selected using G418, subcloned and sorted by flow cytometry to obtain stable cell lines with equal GFP-tubulin expression. Cells were lysed and samples were analyzed by immunoblotting using an antibody against GFP. (**D**) Live cell imaging was used to analyze the time from anaphase onset to abscission, based on the DIC and GFP-tubulin signal for 66 cells per WT clone and 51 cells per K460R clone. Each individual cell is represented by a colored triangle, while the medians with interquartile ranges are shown in black for each set of WT and K460R clone. P values were calculated using the Mann Whitney test. One star represents $p < 0.05$ and three stars $p < 0.0005$.

Journal of Cell Science • Supplementary information

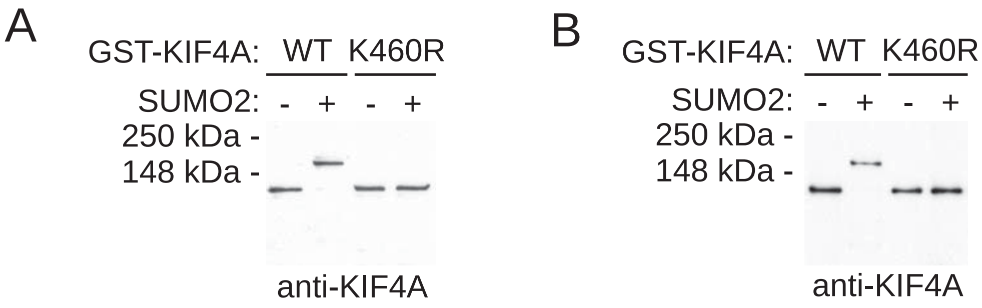

Figure S6 Confirmation of *in vitro* SUMOylation KIF4A before and after lysate incubation. (**A**) Recombinant GST-KIF4A wildtype (WT) or SUMOylation deficient mutant (K460R) were bound to beads and an *in vitro* SUMOylation assay was performed in the absence or presence of SUMO2. Aliquots of the samples were used to verify SUMOylation efficiency by immunoblotting using an antibody against KIF4A. (**B**) The samples described in Supplementary information, Figure 6A were incubated with a U2OS lysate and washed. Prior to sample preparation for mass spectrometry analysis, aliquots of the samples were saved to verify SUMOylation and protein levels by immunoblotting using an antibody against KIF4A. Each experiment was performed at least three times.

Journal of Cell Science • Supplementary information

A

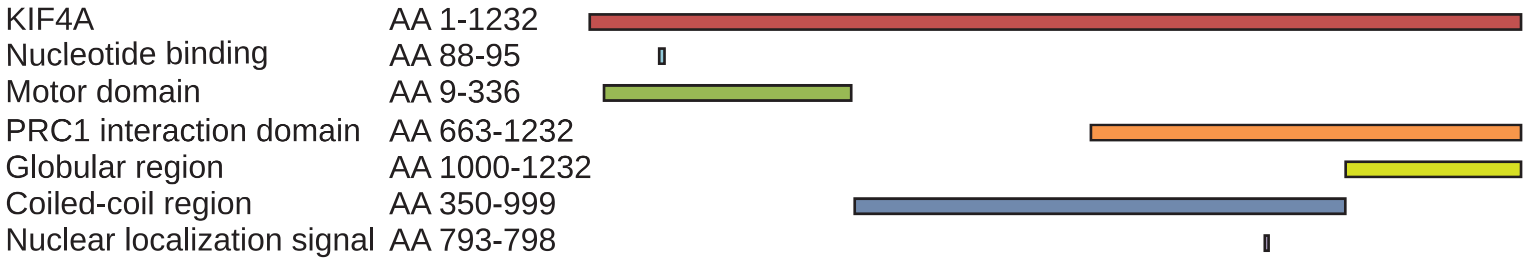

B

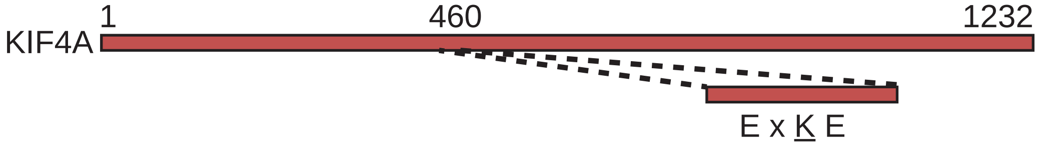


```
Homo sapiens                   428 EKMNAKLEELRQ-------HAACKLDL-QKLVETLEDQELKENVEIICNL 469
Canis lupus familiaris         429 EKMNAKLEELRQ-------HAALKVDL-QKLVETLEDQELKENVEIIRNL 470
Bos taurus                     429 EKMNAKLEELRK-------HAACKVDL-QKLVETLEDQELKENVEIIRNL 470
Mus musculus                   429 EKMNAKLEELRR-------HAACKVDL-QKLVETLEDQELKENIEIICNL 470
Rattus norvegicus              429 EKMNAKLEELRQ-------HAACKVDL-QKLAETLEDQEVKENIEIICNL 470
Gallus gallus                  431 EKMNAKLEQLQQ-------HAVCKLDL-QKLLETVEDEELKENVEVIRNL 472
Xenopus (Silurana) tropicalis  429 EKLASKIEELKQ-------HAACKVDL-QRLVETLEDQELKENVGVILDL 470
Drosophila melanogaster        445 DKLRSHVSELKN-------KLDQREQAQFGNENTNGDNEMRDFSLLVNRV 487
Caenorhabditis elegans         416 SRLEEDKAKLQS--------------MLTDVRNTVLNEEMLDAAEVVRSI 451
Arabidopsis thaliana           445 DKLIMIIESVRNGKSLDEIESCQNEDVGLVNKYVSKIQELEGELLHIKNL 494
```

C

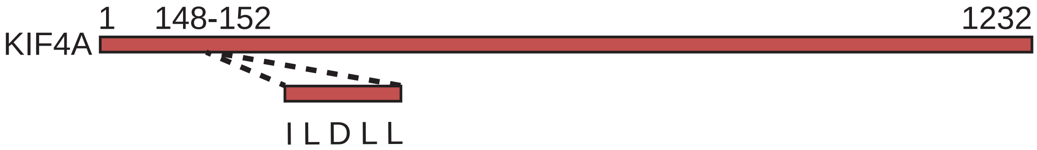


```
Homo sapiens                   139 SYLEIYNEEILDLLCPSRE--------------K--A-QINIREDPKEGI 171
Canis lupus familiaris         139 SYLEIYNEEILDLLCPSRE--------------K--ASQINIREDPKEGI 172
Bos taurus                     139 SYLEIYNEEILDLLCPSRE--------------K--APQINIREDPKEGI 172
Mus musculus                   139 SYLEIYNEEILDLLCSSRE--------------K--ATQINIREDPKEGI 172
Rattus norvegicus              139 SYLEIYNEEILDLLCSSRE--------------K--ASQINIREDPKEGI 172
Gallus gallus                  139 SYLEIYNEDILDLLCSSRE--------------R--SSQISIREDPKEGI 172
Xenopus (Silurana) tropicalis  138 SYLEIYNEEILDLLYAARD--------------K--TNAISIREDPKEGI 171
Drosophila melanogaster        138 SFVELYQEQFYDLFSSKTR--------------D--KATVDIRE-VKNRI 170
Caenorhabditis elegans         136 SMFEVYGDNVYDLLRPDKV------------------KLNVHGDEKN-C 165
Arabidopsis thaliana           134 SFIEIFKEEVFDLLDSNSSALLKNDSGVQAKHTALSRAPIQIRETASGGI 183
```

Figure S7 KIF4A SUMOylation site and SUMO interaction motif conservation. (**A**) A cartoon depicting the localization of known domains and regions in the KIF4A protein. AA, amino acid. (**B**) Cartoon showing the localization of the SUMOylation site, containing lysine 460, in the KIF4A protein and the conservation of this region across the indicated species. (**C**) Cartoon depicting the localization of the SUMO interaction motif (SIM) in the KIF4A protein and the conservation of this region across the indicated species.

Journal of Cell Science • Supplementary information

Table S1 Key of sample names. This sheet explains which sample name belongs to each exact sample measured.

Click here to Download Table S1

Table S2 A list of all peptides identified by Max Quant on the RAW Peptides sheet.

Click here to Download Table S2

Table S3 A list of all protein groups for which peptides were identified by Max Quant on the RAW ProteinGroups sheet.

Click here to Download Table S3

Table S4 A list of proteins for which their binding to KIF4A is significantly affected by SUMOylation. The line separates proteins that were identified to preferentially bind SUMOylated KIF4A (top) from the proteins that favoured binding to non-SUMOylated KIF4A (bottom).

Click here to Download Table S4

Journal of Cell Science • Supplementary information
